# Association Between Unemployment and Mental Disorders: A Narrative Update of the Literature

**DOI:** 10.3390/ijerph21121698

**Published:** 2024-12-19

**Authors:** Andreas G. Franke, Peggy Schmidt, Stefanie Neumann

**Affiliations:** 1 Hochschule der Bundesagentur für Arbeit (HdBA), Seckenheimer Landstr. 16, 68163 Mannheim, Germany; 2 Private Hospital Meiringen, Willigen, 3860 Meiringen, Switzerland; peggy.schmidt@privatklinik-meiringen.ch; 3 European University of Applied Sciences, Werftstr. 5, 18057 Rostock, Germany; s.neumann@eufh-medica.de

**Keywords:** unemployment, long-term unemployment, mental disorders

## Abstract

The relationship between unemployment and mental disorders has been a significant subject of study since at least the Industrial Revolution. However, most data show associations of unemployment and isolated mental disorders, and this study field has been neglected in the last years. Therefore, this narrative review aims to provide an updated overview of the association between unemployment and mental health in general as well as the most prevalent mental disorders. A literature search was conducted using PubMed with the initial search terms “unemployment” and “mental health”. The identified disorders were then used as search terms for a more in-depth search. Two raters screened abstracts and identified the literature containing relevant information. As a main result, it could be demonstrated that regardless of age and sex, there is still a broad association between unemployment and mental well-being in general (e.g., quality of life measure for example by the General Health Questionnaire), suicide attempts, suicide rates, as well as specific psychiatric disorders (substance use disorder, schizophrenia, depression, bipolar disorders, compulsive/obsessive disorders, eating disorders, specific personality disorders, intelligence disorders/impairment, and ADHD). The most significant association was found for affective disorders (depression) and substance use disorders. The association in general was particularly evident for long-term unemployment and mental disorders. Returning to work reduced the prevalence rates of mental disorders significantly. The literature review confirms the results of much older and disease centered studies that unemployment and mental disorders are associated with each other. The main conclusion is that early medical detection and intervention among the mentally ill are as crucial as labor market policy interventions to prevent, avoid, and reduce unemployment.

## 1. Introduction

Research activities regarding the association between unemployment and psychosocial health problems began in the 1930s with “The Unemployed of Marienthal” [1]. Based on the scientific methods of the time, the results indicated a link between mass unemployment caused by the global economic crisis and the emergence of negative psychological and social changes in the affected population [1]. Specifically, unemployed individuals exhibited social, societal, and political inactivity. They became increasingly socially isolated, hardly participated in any social activities, withdrew into the private sphere, became hopeless, neglected themselves, decreased their walking speed, lost their time structure, and showed almost apathetic behavior. The expected uprisings did not occur; instead, there was a stereotypical progression from shock to optimism and active job search, followed by pessimism and distress, fatalism, and adaptation to the unemployed role [2]. In addition to mental and social well-being, physical health also suffered. All of this primarily affected the male population, who had lost their gainful employment. Those affected frequently lamented the “loss of their human dignity as useful members of human society” [1]. The Marienthal study showed for the first time that entering unemployment leads to negative biological, psychological, and social consequences, which impressively reflects the biopsychosocial etiology model [3]. However, the Marienthal study was framed by the situation of mass unemployment. More recent studies conducted more than a decade ago have shown that unemployment is an important factor in many parts of the world and has risen significantly in recent decades [4,5], even if, at least in parts of the European Union, it has fallen again in recent years [6]. Nevertheless, the unemployment rate in the EU is up to 25% (for 15–29-year-olds in Greece), depending on the member state and the group under consideration [7].

The association between unemployment and (biopsychosocial) health problems has been described in various (a) disease-specific and (b) older studies deriving from German-speaking countries (e.g., [8,9,10]) as well as other European countries (e.g., [11]) and ultimately worldwide (e.g., [12,13,14]).

Due to the increasing prevalence and significance of mental disorders, increasing attention is now paid to mental disorders [15]. In the last 30 years, the number of disability-adjusted life-years (DALYs) due to mental illness has risen from 80.8 to 125.3 million. For example, the prevalence rates for depression have been increasing significantly for decades, such that the prevalence rate is currently around 13% and more than 260 million people worldwide are affected by depression [16,17,18,19].

For several years, however, the field of research investigating the association between unemployment and diseases has significantly diminished in activity. In addition, most older studies either provide an overview of overarching aspects of mental health or concentrate on individual disorders.

Identifying a lack of (a) more recent studies and (b) studies about unemployment and the entirety of mental disorders the present review examines the state of research about the association of unemployment and mental health in recent years and the entirety of mental disorders. Furthermore, this narrative review places recent study results in the context of older studies and provides (a) a general overview of the aspects of mental disorders and (b) an overview of the most important mental illnesses in detail together.

## 2. Methods

In order to obtain high significance, this narrative review adopts parts of the methodology of systematic literature reviews.

This review is based on a search in the PubMed database from June to October 2023.

For an initial PubMed search, the search terms “unemployment” and “mental health” were used. Subsequently, the search was narrowed down by using quotation marks (“mental health”).

This was followed by a systematic PubMed literature search for the combination of the search terms “unemployment” and the individual mental disorder identified during the first search. The individual disorders were adapted to the ICD-10 classification. The search terms depression, bipolar disorder, anxiety, schizophrenia, substance use disorders, personality disorder, intelligence disorder/impairment, and compulsive disorder were thus used in combination with the search term “unemployment” and linked by an “and”.

All publications included had to contain the term “unemployment” as the main inclusion criterion as well as one of the abovementioned keywords. In addition, only publications that were written in English or German and that provided information on the aspects of unemployment and mental health following a content review of the abstracts were included in the analysis. The content review was carried out by two of the authors (CN and AGF). Publications identified by both authors as meaningful in terms of content were included; in cases of doubts, there was a discussion about inclusion until a consensus was reached.

## 3. Results

The initial literature search of this narrative review yielded a total of 3488 hits. The number of hits was reduced to a total of 2737 by narrowing down the search as described above. The additional and targeted search for individual mental disorders yielded significantly fewer hits (see Table 1).

Based on the high number of hits, only a limited number of hits could be used in the Results Section to demonstrate specific aspects that are representative of the literature, given in the table below (see Table 1).

### 3.1. Mental Health in General

Overall, the studies showed that unemployment is associated with mental impairment (e.g., [20,21,22]). It has been repeatedly shown that (overall) life conditions and mortality, including suicide rate, parasuicidal behaviors, suicide attempts, and suicidal thoughts, are significantly higher among unemployed people [23,24,25,26], especially suicide rates among 16–34-year-old men during recessions in Great Britain [23]. The suicide attempt rate among young unemployed people is three times higher than among young working people [27]. The abovementioned factors (suicide rate, suicide attempts, and suicidal thoughts) generally correlate with the duration of unemployment: the longer the duration, the higher/more frequent suicidal thoughts/suicide attempts and suicide rate [26,28,29]. Furthermore, the probability of suicide attempts increases with the beginning of unemployment periods, peaks after around five years, and then remains constant [29]. In addition, the association was significantly more pronounced in men (compared to women) [24,30]. Regaining employment led to a significant reduction in suicidal thoughts [25,26]. An international meta-analysis across 20 countries was able to show that in the healthy population as a whole (without the presence of mental disorders), the suicide rate is significantly higher in the case of financial stress and unemployment [31]. A German study was able to show that the life expectancy of people in regions with high unemployment rates is significantly lower; among unemployed men, mortality in the year following an increase in the unemployment rate was 6% higher than during the same period in the previous year. These correlations could be broken down to the district level [32]. The General Health Questionnaire (GHQ) was often used to describe mental health states, in which unemployed people achieved significantly lower scores in several studies [26,28,33]. Warr and Jackson demonstrated that GHQ scores decreased three months after becoming unemployed (against a control group) and stabilized later [33]. These differences after a period of three months was confirmed by Lahelma and colleagues; in contrast, they showed that 15 months after the beginning of unemployment 49% of the unemployed compared to 16% of an employed control showed low GHQ scores [28]. Reobtaining employment led to a significant increase in mental well-being and especially a significantly higher GHQ score [28]; the effect was more pronounced in men than in women [26,28,33]. If women are grouped into married vs. unmarried women and with vs. without children, the abovementioned effect in unmarried women without children is most similar to the effect in men [26]. Apart from this, men in higher professional positions in particular benefit from reobtaining employment in terms of overall health status, especially at an advanced age [34]. In addition, health behavior also changes significantly at the beginning of the unemployment period [35]. Mental well-being and health behavior are more significantly reduced by unemployment in people with a high socioeconomic status (SES) (before entering unemployment) than in people with a low SES [35]. Apart from this, there is a correlation between experiences of distress and unemployment: 17 out of 28 studies demonstrated that unemployment increases psychological distress; the latter in turn increased drug use (see below) [36]. This correlation is less pronounced in women. However, unemployment (over a six months period) also leads to increased (di-)stress levels (using the Hopkins Symptoms Checklist) in young adults regardless of sex [37]; an effect of unemployment on mental health was especially found in all cohort studies that did not control for confounders [20]. In addition, entering unemployment for more than 3 months during a 2-year period leads to a so-called “disrupted identity” as one of the main outcomes of qualitative interviews [38]. Unemployment also reduces overall life satisfaction (in young people) [20]. The correlation between lower mental well-being (i.e., demonstrated by a low GHQ score) and unemployment is generally applicable to all age groups, including those under 25 years [20,26]. The duration of unemployment correlates with the degree of mental impairment and affective well-being [26,39]. It was shown that mental well-being decreased during the first six months of unemployment and then reached a plateau. Young unemployed adults also have a fourfold increased risk of being diagnosed with any mental disease [27]. However, unemployment has “long-term effects”, too: it has been shown that unemployment significantly increases the probability of a lower quality of life and mental impairment (using the Psychological Problem Index (PPI) and self-designed instruments) in later life—even many years after the unemployment has taken place [20,40]. In addition, it has been shown that even many years after being unemployed, there is a significantly higher risk of suicide (demonstrated by suicide rates) and impaired mental health (i.e., using PPI and semi-structured interviews) among those who were formerly affected by unemployment [38,40,41]. However, not only unemployment but also an unstable type of employment significantly impairs mental health, which was shown in 16 out of 24 studies described by Kim and von dem Knesebeck [42].

### 3.2. Substance Abuse and Substance Dependence Disorders (F1)

Substance abuse is characterized by a pattern of psychoactive substance use leading to damage to health that may be physically or mentally disruptive [43]. According to the ICD-10, substance dependence is characterized by a cluster of behavioral, cognitive, and physiological phenomena that develop after repeated substance use. It typically includes a strong desire to use the respective psychoactive substance, difficulties in controlling its use, persisting in its use in spite of harmful consequences, a higher priority of using the respective drug, increased tolerance, and sometimes physical withdrawal symptoms in case of abstinence [43]. Psychoactive substances (drugs) are alcohol, tobacco, opioids, cannabinoids, sedatives/hypnotics, cocaine, stimulants, and volatile solvents [43].

Substance abuse and substance dependence disorders can be summarized as substance use disorders (SUDs). It should be noted that the prevalence of SUDs is significantly higher among unemployed people in general than among employed people (e.g., [44,45]). The vast majority of studies on SUDs and unemployment focus on alcohol (e.g., [46,47,48]), followed by studies on tobacco use (e.g., [12,49]) and cannabis (e.g., [50,51]); studies on illicit drugs such as cocaine and opioids are much rarer (e.g., [52,53,54]). However, nearly all studies show significant associations between unemployment and substance use or even SUDs involving various psychotropic substances [55]. Some specific findings show that the amount and frequency of alcohol consumption as well as alcohol-associated death rates are significantly higher and that consumption patterns are “more severe” among unemployed people [56,57,58]. The longer the unemployment lasts, the more alcohol-associated symptoms occur (14% more alcohol-associated symptoms per year) [59]. In addition, there are significant associations between economic recessions, binge drinking, and SUDs [30,45].

### 3.3. Disorders from the Schizophrenic Spectrum (F2)

In general, schizophrenic disorders are characterized by fundamental and characteristic distortions in thinking and perception and affects that are inappropriate or blunted. Certain cognitive deficits may evolve in the course of time. The most important psychopathological phenomena include thought echo, thought insertion or withdrawal, thought broadcasting, delusional perception and delusions of control, influence or passivity, hallucinatory voices, thought disorders, and so-called negative symptoms with decreased internal drive and affect flattening [43].

There are only a few studies on the association between unemployment and schizophrenia. A systematic review shows that early stages of schizophrenia are already associated with a significantly higher unemployment rate, which is between 10% and almost 90% of those affected (compared to a healthy control group) [60]. In addition, early-stage schizophrenia is also associated with higher mortality, psychiatric comorbidities such as SUD or depression, and a low level of social functioning [60]. Although review studies mostly do not contain sufficient information about specific instruments used (e.g., Positive and Negative Symptom Scale, PANSS, etc.), specific studies these reviews are based on show that PANSS scores are significantly higher among unemployed people suffering from schizophrenia (n = 19 employed: 29.9 ± 10.8 vs. N = 81 unemployed: 43.1 ± 13.8 [61]). Furthermore, people with an exceptionally high risk of developing schizophrenia were significantly more frequently affected by unemployment despite not yet having developed schizophrenia [62]. Other studies point to the association of schizophrenia and unemployment via indirect indicators (e.g., loss of productivity, early retirement, early death, homelessness, and cognitive impairment) (e.g., [63,64]). On the other hand, studies (i.e., using the Structured Clinical Interview, SKID, for diagnosing schizophrenia) have also indicated that unemployment (in addition to other risk factors such as low income, low educational level, and family history of mental illness) is a risk factor for the development of schizophrenia (e.g., [65]). A case–control study showed that emotional and psychological well-being (measured by the Mental Health Continuum, MHC) was significantly lower in the group of unemployed people with schizophrenia (MHC emot. well-being: 5.95; MHC psych. well-being: M = 11.8) compared to the employed group (MHC emot. well-being: 7.8; MHC psych. well-being: M = 15.8; *p* < 0.01) [66]. In addition, a longitudinal study showed that a ten-year course of schizophrenia is strongly associated with unemployment [67]. In a comparison of patients diagnosed with schizophrenia versus bipolar affective disorders using SCID, medical records, and interviews according to the DSM over 20 years, patients with a diagnosis of bipolar affective disorders were significantly less likely to be unemployed than schizophrenics [68].

### 3.4. Affective Disorders (F3)

Affective or mood disorders are characterized by fundamental disturbances regarding affect (from depression to elation) accompanied by changes in the overall level of activity [43].

Numerous studies indicate an association between mood disorders and unemployment (e.g., [30]). For example, Power and colleagues found a twofold increased risk of lifetime depressive disorders to be associated with unemployment states (OR = 2.9, CI = 0.9–4.2, *p* = 0.051) [27]. Other studies have confirmed these results: an up to twofold increased risk of developing clinically relevant depressive symptoms, and even higher scores by the use of the Patient Health Questionnaire (PHQ) among unemployed people who were 50 years and older [9]; in particular, long-term unemployment leads to a significant risk of developing depression [9,21,22,38]. For example, using the Beck Depression Inventory (BDI), a significant difference (*p* < 0.001) was found between long-term unemployment (14.2 ± 9.5) and short-term unemployment (10.1 ± 8.8) [69]. Large meta-analyses showed that the prevalence of depression increased significantly at the beginning of the unemployment period. Upon re-entry into working life, there was a significant decrease [13,21]. In a large-scale study using, i.e., the Goldberg Depression Scale (GDS), among 2.400 participants demonstrated unemployment and underemployment to be significant predictors of depression [70]. A meta-analysis revealed that prevalence rates of major depressive disorder (MDD) diagnosed according to DSM-IV criteria are particularly high among the unemployed (16%, OR 1.88, 95% CI [1.57, 2.25]) and particularly high in men compared to in women (men: OR 2.27, 95% CI [1.76, 2.93]; women: 1.62, 95% CI [1.40, 1.87]) [71]. Unemployed men suffer significantly more frequently from depressive disorders and dysthymia than unemployed women [71,72]. Furthermore, bipolar affective disorders correlate with unemployment (e.g., [73]). There is an association between cognitive deficits and the course of the illness (hospitalization frequency, etc.) of those who are affected [73]. Overall, an average of 60% of those affected by bipolar affective disorders are unemployed and 88% report workplace-related problems [74]. Larger longitudinal studies have repeatedly shown the association between unemployment and cognitive impairment using multiple neurocognitive tests (i.e., Repeatable Battery for the Assessment of Neuropsychological Status, Wechsler Intelligence Scale, Trail Making Test) in people with bipolar affective disorders diagnosed by Brief Psychiatric Rating Scale, the Hamilton Depression Scale, and the Young Mania Rating Scale [74,75]. Patients who are affected by bipolar disorders have a significantly higher risk (sevenfold) of being absent from work due to their illness [74,76,77].

### 3.5. Anxiety and Obsessive–Compulsive Disorders (F4)

Anxiety disorders are dominated by anxiety as the major symptom, which is restricted to specific environmental situations or generalized. The specific situations are characteristically avoided or endured with dread. The patient’s concern may be focused on individual symptoms like palpitations or feeling faint and is often associated with secondary fears of dying, losing control, or going mad [43]. Obsessive–compulsive disorders are dominated by recurrent obsessional thoughts or compulsive acts. Obsessional thoughts are ideas, images, or impulses that enter the patient’s mind again and again in a stereotyped form. They are almost invariably distressing and the patient often tries, unsuccessfully, to resist them [43].

Together with depressive disorders, anxiety disorders have the highest prevalence rates in the population [78]. Under conditions of unemployment, the prevalence of anxiety disorders is significantly higher compared to the working population [22,79,80]. In particular, this is especially valid for long-term unemployment (Hospital Anxiety and Depression Scale, HADS > 8) [79]. Numerous studies indicate a connection between anxiety disorders and unemployment (e.g., [30]). A large meta-analysis recorded a significant increase in the prevalence of anxiety disorders upon entering unemployment periods; upon re-entry into employment, a significant decrease in anxiety disorders could be observed [13,21]. Approximately 47% of the long-term unemployed show symptoms of an anxiety disorder [81]; those in full-time employment had a significantly lower intensity of symptoms of anxiety than unemployed people [81]. Young unemployed adults have a twofold increased risk of developing anxiety disorders (OR = 2.63, CI = 1.13–6.06, *p* = 0.023) [27].

Unemployed men are significantly more likely to have panic disorders and phobias compared to women [72]. In addition, qualitative interview studies have shown that the experience of mass unemployment (e.g., in economic crises) leads more frequently to anxiety [38,82]. As with schizophrenia and affective disorders, the severity and persistence of anxiety disorders and their impact on the employment relationship and absence from work due to the diseases are particularly pronounced [83]. Studies on obsessive–compulsive disorder (OCD) are very rare in connection with unemployment. Nevertheless, isolated findings show that OCD is associated with unemployment, too (e.g., [84,85,86]); using the Yale–Brown Obsessive–Compulsive Scale, unemployed OCD patients appear to have a significantly poorer outcome in (psycho-)therapy (cognitive behavioral therapy). A large epidemiological study of n = 16,267 16–64-year-old OCD patients versus n = 157,176 healthy controls showed marginalizing effects of those affected on the labor market as well as a significantly increased risk of long-term unemployment (adjusted hazard ratio = 1.72 (95% CI 1.63–1.82) [87]; the absence of comorbidities has hardly any effect, so OCDs are causally in the foreground.

### 3.6. Eating Disorders (F5)

The most frequent types of eating disorder are anorexia nervosa (characterized by deliberate weight loss, induced and sustained by the patient, most commonly occurring in adolescent girls and young women) and bulimia nervosa (characterized by repeated bouts of overeating and an excessive preoccupation with the control of body weight, leading to a pattern of overeating followed by vomiting or use of purgatives).

According to the assessment of a previous study, there are hardly any significant studies regarding eating disorders and unemployment [88]. Using multiple self-rating questionnaires, a recent study indicates that although eating disorders are not significantly associated with employment status, eating disorders are associated with lower professional performance [89]. However, using DSM-IV criteria and the Eating Disorder Examination Questionnaire, another recent study showed that there was indeed an association between anorexia nervosa and unemployment derived from high scores by the Brief Disability Questionnaire [90], but that this did not appear to persist significantly over the long term (10 years after diagnosis) (unemployment among anorexia nervosa: 5% vs. healthy controls: 4%, *p* = 0.62) [91]. There is evidence, however, that binge eating disorders are associated with parental unemployment in addition to other factors [92,93]. Further, impactful studies cannot be found.

### 3.7. Personality Disorders (F6)

Personality disorders are severe disturbances in the personality and behavioral tendencies of the individual usually involving several areas of the personality; they are nearly always associated with considerable personal distress and social disruption, usually manifesting in childhood/adolescence and continuing throughout adulthood [43].

It is striking that many of the studies reported comorbidities with personality disorders (e.g., depression, bipolar affective disorder) (e.g., [94]). Antisocial personality disorders are highly associated with unemployment (e.g., [95]). In addition, using multiple assessment tools (e.g., SCID, Multisource Assessment of Personality Pathology, NEO Personality Inventory—Revised, etc.) it shows that there is an association between borderline personality disorder (BPD) and unemployment [96,97].

The characteristics of BPD and stigmatization serve as significant mediating factors for unemployment in BPD patients [98]. Beyond that, unemployment was also found to be significantly associated with BPD when bipolar affective disorders co-occurred [99].

### 3.8. Mental Retardation/Intelligence Disorders (F7)

Mental retardation/intelligence disorders describe a condition of arrested or incomplete development of the mind, which is especially characterized by impairment of skills that belong to intelligence (i.e., cognitive, language, etc.). Degrees of mental retardation are conventionally estimated by standardized intelligence tests (mild retardation: approximate IQ range of 50 to 69, etc.) [43].

Cognitive deficits are generally associated with unemployment. However, there are hardly any studies on intelligence disorders as defined by the ICD. A longitudinal study using standardized IQ testing (Wechsler Intelligence Scale—Revised) over 25 years showed significantly (*p* < 0.001) that a low IQ (<94) compared to a higher one measured at the age of eight/nine years is associated with later unemployment [100]. An earlier study conducted in Germany identifies a correlation between learning disabilities and unemployment [101]. A smaller German study suggests an association between low IQ and receipt of disability income [102].

### 3.9. ADHD (F9)

Attention deficit/hyperactivity disorder (ADHD) belongs to the group of disorders characterized by an early onset (<6 years), a lack of persistence in activities that require cognitive involvement, and a tendency to move from one activity to another without completing any one, together with disorganized, ill-regulated, and excessive activity [43].

Patients with ADHD exhibit significantly lower job-related performance, job stability, and higher rates of unemployment compared to non-ADHD individuals. This is supported by substantial evidence from both short-term and long-term studies across various age groups (ADHD diagnosis associated with unemployment: Erskine et al.: OR = 2.0, 1.0–3.9; Gjerman et al.: 22.2% of ADHD patients had ordinary work compared to 72% in general population; Jangmo et al.: ADHD patients 12.2 (11.9–12.5) more days of unemployment annually) (e.g., [103,104,105,106,107,108]). With appropriate drug treatment (at least 2 years), the unemployment rate among those who are affected by ADHD could be significantly reduced (10% lower risk of long-term unemployment in the following year, adjusted RR 0.90 [95% CI, 0.87–0.95]). However, this is valid for women and not for men. Furthermore, the longer the treatment, the lower the risks of unemployment states in women (RR for medication of 1–6 months, 0.86 [95% CI, 0.78–0.95]; RR for medication use of 18–24 months, 0.72 [95% CI, 0.58–0.90]; *p* < 0.001) [109].

The following table (Table 2) offers an overview of the content of the studies this review is based on.

## 4. Discussion

The literature search showed and confirmed a significant association between unemployment and mental health in general as well as the majority of major ICD-10-listed disorders by the literature from recent years and decades.

Regarding the aspect of unemployment decreasing mental health aspects in general, some aspects must be discussed before focusing on specific diseases. For example, in their study, Arena and colleagues [38] discussed factors that represent the relationship between unemployment and mental health. Against the background of the latent deprivation model, the negative influence on mental health is not surprising, as the loss of employment also means the loss of sense and identity, two important psychosocial functions [38]. As the duration of unemployment and age increase, along with the combination of these factors, insecurities and fears regarding one’s self-worth and capabilities intensify, making it more challenging to secure or regain employment. This issue exacerbates with prolonged unemployment [38]. This can be understood from the perspective of “work” and the ability to work as a key aspect of considering oneself as an acceptable member of society; however, it is merely a socially constructed perspective due to socialization. Unemployed people feel stigmatized and are excluded from society [38]. Feelings of failure arise and a reduction in self-esteem may be the consequence. Younger unemployed people may miss sufficient progress in their development, especially employment development as a normative development task of establishing themselves in the world of the workforce. This must be considered against the background of socialization and the development of one’s own identity being influenced by a lack of work [40]. It was shown that older employees often feel ashamed because they did not fulfil the normative societal expectation of being an established, independent, and productive member of society [38].

Furthermore, the search for financial support was perceived as very unpleasant, triggering negative self-assessments, e.g., the feeling of being a burden to others. This may lead to a conflict between the desire to be independent and to take care of oneself and the reality of being a burden for others. In this context, unsuccessful job application processes, perceived as personal devaluation, may result in the avoidance of future applications, thereby diminishing motivation for subsequent job searches. These repeated experiences with the feeling of helplessness and loss of control have a negative influence on general health (theory of learned helplessness by Seligman 1975 [110]; transactional stress model according to Lazarus and Folkman 1984 [111]), which is underlined by Herbig and colleagues [9]. Other factors that have an unfavorable effect on mental health in connection with unemployment are, for example, the lack of income. This in turn creates stress and makes a healthy lifestyle more difficult [9].

Beyond that, employment has several functions such as contact with others, integration (in groups), (social) activities, experiencing meaningfulness, daytime structure, personal status, finding and establishing identity shaped by the respective job, etc. [9]. In the case of unemployment, these functions are lacking in unemployed people. This demonstrates the holistic role of the environment combining the economic function of employment with the psychosocial function [38]. In addition, there are further aspects of losing one’s job, such as unpredictability or perceived injustice, that also appear to have a significant influence on mental health [112].

Regarding suicidality, cohort effects are discussed [41]: In the case of unemployment, men are more likely to attempt suicide. The link between a decline in well-being during unemployment also appears to be more pronounced in men [26]. Owen and colleagues [26] explain this result by the fact that women, in contrast to men, traditionally have another way of identifying with the role of a housewife if lacking the role of nurturer of the family. However, a prerequisite for this difference seemed to be the fact that women were in a stable partnership with clear traditional roles. Higher mortality rates among unemployed men in the year following an increase in the unemployment rate [32] seem to be associated with the abovementioned aspect. Furthermore, this might be due to higher deviant behaviors such as substance abuse and aggression as well as an increased suicidal rate among men [95].

Beyond the general mental health aspects previously discussed, it is essential to focus on specific disorders classified under the ICD-10.

Concerning SUDs (F1), it is quite plausible that psychoactive substances are used in the sense of self-medication—even if SUDs belong to the group of mental disorders; this is particularly important among unemployed people with the abovementioned psychological problems and psychological stress that are suffered from [36,44,45]. On the other hand, people who already use psychoactive substances are more likely to lose their jobs [44,45]. One possible reason that is often discussed is the rapid onset of mental disorders when unemployment occurs, which is in line with unfavorable health and substance use behavior [44]. The relationship between SUDs and unemployment reveals the vitious circle of the relationship as well as the interchangeability of origin and consequence.

Regarding psychotic disorders (F2), the first onset, especially in schizophrenia, occurs during the (vocational) training period around the age of twenty [65]. This may lead to significant difficulties in working life. Furthermore, patients with early schizophrenia have poorer social skills [60], which makes professional integration even more difficult. In addition, there are limitations in key functional areas such as cognition and low physical fitness [68] leading to limited functioning in professional life. This lack of functioning has been already observed in childhood [62]. This is in line with the tendency of lower social functioning in the chronic progression of psychotic disorders [60]. Furthermore, this goes along with increased criminality and possible convictions, deviant behavior, and stigmatization [113]; long periods of hospitalization occur as a result of the disease or its symptoms [60]. Conversely, it must be stated that unemployment triggers and predicts the occurrence of psychotic disorders in the case of corresponding vulnerability [62]. One possible explanation is a depressive processing of job loss and resulting stress.

Absenteeism and frequent hospitalization can also promote unemployment and make it more difficult to return to work in the case of affective disorders (F3). From a psychotherapeutic perspective, it should be understandable that experiences such as an increased level of distress as well as experiences of “disrupted identity” and “disrupted direction”, the loss of the feeling of being needed, and the loss of vision for the future [20,26,36,37,38] can lead to the development of depression. This can be substantiated by taking into account the findings from the theory of learned helplessness [110]. The gender difference, with a significantly higher prevalence rate of depression and suicidality among men, may be due to traditional roles [26]. Workplace-related problems that occurred in the context of bipolar disorder could be explained by the fact that patients did not achieve functional recovery during treatment despite the absence of affective symptoms. In contrast, a better course of the disease was observed in the case of positive occupational experiences and thus a faster remission of bipolar affective disorder [73]. The focus of treatment is therefore on functional recovery rather than purely affective symptom relief, as was previously the case [73].

Considering the concept of missing self-confidence and a lower degree of trust in one’s capabilities (see above), it is not surprising that unemployed people suffering from anxiety and OCD (F4) have lower self-esteem [9,22]. Fears about the future also play a major role (e.g., due to loss of earnings, rejected job applications, loss of confidence in one’s capabilities). Regarding daily functioning, patients with severe OCD are at a severe disadvantage in the labor market compared to healthy people. In comparison to other mental disorders, they are in second place behind schizophrenia and non-affective psychoses in terms of this observed disadvantage [87]. The reasons for this are long periods of absence due to illness or, ultimately, disability pensions [87]. People who receive a disability pension no longer appear in unemployment statistics; this must be considered as a sign of the severity of symptoms and the incapability to meet the criteria of working performance of the labor market.

In the case of eating disorders (F4), Tan and colleagues [90] assume a connection between lower employment rates and lower performance due to low body weight in anorexia nervosa [90]. However, the patients themselves do not see their low body weight as a disadvantage. They even deny having low body weight, which is a symptom of anorexia nervosa [90]. Denial of illness is at least partly responsible for the high number of treatment dropouts, although it should be kept in mind that high treatment dropout rates also occur in other forms of eating disorders [92]. At best, a partial ability to work can be achieved. However, this is based on the significant instability of the disease, which makes stable and long-term employment more difficult [91]. Even if the long recovery process leads to an increase in the disease, many studies nevertheless point to serious socioeconomic consequences even after overcoming anorexia [91]. However, as already mentioned, only a few studies deal with the connection between eating disorders and unemployment.

Personality disorders include a broad spectrum of disorders with varying degrees of severity. This means that further differentiation is mandatory. Cruitt and colleagues [95] posit that for individuals with symptoms of borderline personality disorder (BPD), the loss of employment—and consequently the loss of structure, social contacts, and self-confidence—has detrimental health effects. Unemployment may lead to an increase in the negative effects of borderline personality disorders, and, in turn, employment may lead to an improvement in functioning (positive self-image, identity) [96,97]. This stresses the importance of employment to reduce BPS symptoms and to increase social functioning. However, frequent problems in keeping a job for BPS patients are based on disease-specific symptoms such as self-harming behavior, unfavorable behavior, etc. Furthermore, BPS patients often show reduced work performance capability [96]. Concerning antisocial personality disorder (ASPD), numerous issues such as criminal behavior, substance abuse, homelessness, and relationship difficulties contribute to unemployment or prolonged joblessness [95]. Personality disorders often contribute to the development of comorbid conditions that are associated with unemployment. Additionally, personality disorders negatively impact the progression of these comorbid conditions, exacerbating the overall problem [95].

Focusing on intelligence disorders, there is an association between a lower IQ and criminality, drug use, and mental health problems in general [100]. As already stated, these variables favor unemployment (e.g., [95,113]). However, it is important to note that low intelligence in the absence of early behavioral problems and social disadvantage was not associated with a demonstrable increase in these problems.

Failures in school development associated with ADHD, compensated using psychoactive substances and, as a possible consequence, a lack of vocational training can promote unemployment. However, this may favor the development of comorbid disorders, which then have an additional unfavorable effect on the ability to work [103]. In turn, during employment, the occupational stress of ADHD symptoms is very high [107], which may favor stress-related disorders and is associated with absences from work and dismissals [107].

### Limitations

The significance of this article is limited by some aspects that are worth mentioning. The search was limited to PubMed and the search terms listed above. This may have led to the fact that some of the literature could not be found. Using more databases would have led to more research results. However, PubMed can be considered a primarily biomedical database with a strong focus on the health-related literature including studies on mental health and social determinants like unemployment. PubMed offers comprehensive access to peer-reviewed articles from health and psychology journals that focus on the mental health impacts of unemployment. Of course, unemployment is an economic and/or sociological topic. Nevertheless, it has broad and severe psychological and health-related consequences that are core aspects in PubMed. Furthermore, PubMed indexes a wide range of clinical, epidemiological, and public health studies directly related to how unemployment affects mental health. Beyond that, the articles indexed in PubMed are sometimes considered more credible than those in broader or less curated databases. The use of other databases such as PsycINFO, Scopus, Web of Science, or Embase might be more interdisciplinary and may cover more fields such as economics, sociology, and policy. Therefore, significant health- or even (mental) disease-specific research results beyond those indexed in PubMed were not expected from searching other databases. Nevertheless, the use of only one database may be the main limiting aspect of this study.

Apart from the choice of database, there are qualitative and, above all, methodological differences between the studies included in this review. For example, in some studies, the diagnoses were assigned clinically, while in others, they were determined using standardized questions. This makes the results difficult to integrate into each other and interpret commonly. Another important aspect is that there is no information on comorbidities in many studies because they have not been monitored or have been excluded from further consideration. It is therefore often unclear whether comorbidities were present and what role they may play. Beyond that, the durations of unemployment were not consistent. These aspects may have led to certain bias.

Another aspect limiting the significance of this article is the choice of search terms as well as the choice of studies. The choice of the abovementioned search terms may have led to the fact that not all relevant research results could be identified. Beyond that, the fact of using two raters screening the abstracts and deciding on the in-/exclusion of articles may have increased the number of meaningful literature hits. This may have helped to reduce the subjectivity of the raters, however, bearing the problem of an inter-rater reliability.

Another important aspect is the character of this study. This manuscript presents results as a narrative review with a broader question. However, to strengthen the narrative results, elements of a systematic review were used. Nevertheless, this review is not a systematic review and does not contain specific statistical tests (e.g., Kohen’s kappa, G*power analysis), which may limit the significance of the content.

## 5. Conclusions

Across all disorders, their specific symptoms and psychosocial consequences, along with specific disorders, were found to be a mediating factor in becoming unemployed. In addition, the psychosocial aspects interact with each other and thus may exacerbate the overall situation. Beyond that, mental disorders in childhood and adolescence may remain undetected. Even though most mental disorders from childhood and young adulthood remit by the age of 20, economic effects are already noticeable, e.g., due to poorer school performance and performance during vocational training periods. Other diseases and their specific symptoms begin at later stages and may remain apparent, which makes it more difficult to stay employed.

This review was able to confirm results of earlier reviews of a scientific field that has been neglected in the last years and still is. It shows that most recent studies confirm the association between unemployment and specific mental disorders as well as mental problems in general.

The significant association between unemployment and mental disorders is still valid and relevant. Researchers should be and stay well aware of this association—especially in times of rising unemployment rates, a worsening in economic situation, and increasing importance of mental health. The data have important implications, emphasizing the importance of prevention and health promotion in general as well as the importance of paying attention to psychiatric symptoms and initiating therapy in the early stages. Moreover, this review also serves to encourage active labor market policy interventions that have the potential to prevent mental disorders, thus preventing, avoiding, and reducing unemployment.

## Figures and Tables

**Table 1:** Numerical results of the literature search.

Disorders According ICD-10: Fx	Hits
Unemployment + substance use disorder (ICD-10: F1)	N = 1398
Unemployment + schizophrenia (ICD-10: F2)	N = 383
Unemployment + mental health + depression (ICD-10: F3)	N = 1131
Unemployment + bipolar disorder (ICD-10: F3)	N = 155
Unemployment + anxiety disorder (ICD-10: F4)	N = 681
Unemployment + compulsive disorder (ICD-10: F4) Unemployment + obsessive disorder (ICD-10: F4)	N = 29 N = 36
Unemployment + eating disorder (ICD-10: F5)	N = 28
Unemployment + personality disorder (ICD-10: F6)	N = 312
Unemployment + mental retardation (ICD-10: F7) Unemployment + Intelligence disorder (ICD-10: F7) Unemployment + intelligence impairment (ICD-10: F7)	N = 98 N = 127 N = 24
Unemployment + attention deficit/hyperactivity disorder (ICD-10: F9)	N = 70

**Table 2:** Summary of the results on the relationship between unemployment and mental health problems with key findings and cited references.

Category	Findings	Key Details	Cited References
General mental health impact	Unemployment is broadly associated with reduced mental well-being, including increased suicidal thoughts and distress.	Effects intensify with longer unemployment; reemployment significantly improves outcomes. GHQ scores drop after job loss and improve upon reemployment. Gender differences exist, with men often more affected.	[20,21,22,23,24,25,26,27,28,29,30,31,32,33,34,35,36,37,38,39,40,41,42]
Substance use disorders (F1)	Unemployed individuals show higher prevalence of SUDs, including alcohol, tobacco, and cannabis use.	Severity of substance use often increases with unemployment duration. Alcohol abuse is most studied; binge drinking and drug use are also common. Economic recessions exacerbate SUD prevalence.	[43,44,45,46,47,48,49,50,51,52,53,54,55,56,57,58,59]
Schizophrenia (F2)	High unemployment rates among individuals with schizophrenia due to cognitive and social impairments.	Early schizophrenia is associated with unemployment rates of 10–90%. Risk factors include lower social skills, comorbid SUDs, and stigma. Unemployment can also predict onset in vulnerable individuals.	[43,60,61,62,63,64,65,66,67,68]
Affective disorders (F3)	Depression and bipolar disorders are significantly associated with unemployment, especially long-term unemployment.	Depression risk increases 2–3 times with unemployment. Long-term unemployed show higher BDI scores. Reemployment improves mental health. Bipolar disorder leads to absenteeism and workplace challenges.	[9,13,21,22,27,30,38,43,69,70,71,72,73,74,75,76,77]
Anxiety disorders (F4)	Anxiety disorders are more common among the unemployed, with severity increasing during long-term unemployment.	Long-term unemployed (HADS > 8) show higher anxiety. Young unemployed adults have a doubled risk. Reemployment significantly reduces symptoms.	[13,21,22,27,30,38,43,72,78,79,80,81,82,83,84,85,86,87]
Eating disorders (F5)	Limited studies suggest unemployment impacts anorexia nervosa outcomes and professional performance.	Recovery from anorexia does not always improve employment outcomes. Parental unemployment may influence binge eating in adolescents. Evidence is sparse.	[88,89,90,91,92,93]
Personality disorders (F6)	Unemployment correlates with BPD and ASPD due to behavioral challenges and stigma.	BPD symptoms like impulsivity and self-harm hinder job retention. Employment improves functioning and identity in BPD patients. ASPD often involves SUD and criminal behavior.	[43,94,95,96,97,98,99]
Mental retardation/intelligence disorders (F7)	Lower IQ is strongly linked to higher unemployment rates and social challenges.	Individuals with low IQ (<94) are more likely to face unemployment. Intellectual impairments correlate with receipt of disability income	[43,100,101,102]
ADHD (F9)	ADHD increases unemployment risk due to job instability and lower performance.	ADHD patients are twice as likely to face unemployment. Long-term treatment reduces unemployment rates, especially in women.	[43,103,104,105,106,107,108,109]

## Data Availability

Data search was performed in PubMed. All relevant publications this review is based on were stored by the authors on (private) hard discs.

## Abbreviations

ADHD	attention deficit/hyperactivity disorder
ASPD	antisocial personality disorder
BDI	Beck Depression Inventory
BPD	borderline personality disorder
GDS	Goldberg Depression Scale
GHQ	General Health Questionnaire
HADS	Hospital Anxiety and Depression Scale
MDD	major depressive disorder
MHC	Mental Health Continuum
OCD	obsessive–compulsive disorder
PANSS	Positive and Negative Symptom Scale
PHQ	Patient Health Questionnaire
PPI	Psychological Problem Index
SES	socioeconomic status
SKID	Structured Clinical Interview
SUD	substance use disorder
